# Organic solvent-free micellar liquid chromatographic determination of amoxicillin, chlorzoxazone, and ibuprofen in pharmaceutical and biological matrices

**DOI:** 10.1038/s41598-026-64529-1

**Published:** 2026-08-07

**Authors:** Naglaa A. Kabil, Mariam Zakaria, Hawa M. Khalil, Sara Abdulwahab

**Affiliations:** Department of Pharmaceutical Analytical Chemistry, Faculty of Pharmacy, Zagazig University, Zagazig, Egypt

**Keywords:** Amoxicillin, Chlorzoxazone, Ibuprofen, MLC, SDS, Biological techniques, Chemistry, Drug discovery

## Abstract

Athletes are frequently prescribed combination therapies to manage bacterial infections and musculoskeletal injuries, often involving amoxicillin (AMX), ibuprofen (IBU), and chlorzoxazone (CHL). However, their co-administration presents analytical challenges due to the structural diversity of these drugs within complex matrices. Simultaneous quantification of these agents is particularly demanding in the context of pharmacokinetic studies, therapeutic drug monitoring, and pharmaceutical quality control, where sensitivity, selectivity, and matrix compatibility are essential. To address this challenge, the present study introduces a rapid, robust micellar liquid chromatographic (MLC) method demonstrating an environmentally favorable profile relative to conventional acetonitrile-based RP-HPLC comparators, for the simultaneous determination of AMX, CHL, and IBU in their combined pharmaceutical dosage forms, and human plasma samples. The method utilizes a short RP-C18 column i.e., 75 mm, with UV detection at 220 nm, achieving complete separation of all three analytes in under 6 min using a low impact micellar mobile phase composed of sodium lauryl sulfate (SDS), Brij-35, and potassium dihydrogen phosphate at pH 3.0. Method validation was performed according to ICH guidelines, demonstrating linearity (R² > 0.999), accuracy (98.6–101.1%), adequate sensitivity (LOD: 0.44–0.74 µg/mL; LOQ: 1.32–2.25 µg/mL), and excellent baseline resolution between adjacent peaks (Rs > 4.5) across the concentration range of 2.5–25 µg/mL for all three analytes. Environmental sustainability was quantitatively assessed; the proposed method achieved an Eco-scale score of 87, compared to 68–78 for previously reported RP-HPLC methods, providing a quantitative basis for the green chemistry comparison. In alignment with green chemistry principles, the method’s environmental sustainability was further evaluated using multiple metrics including the Analytical Eco-Scale, GAPI, AGREE, BAGI, and RGB12 tools. The collective results indicate that the proposed method’s environmental profile compares favorably with previously reported RP-HPLC procedures, though it is acknowledged that the applied greenness metrics do not fully capture potential environmental persistence of non-ionic surfactants such as Brij-35 at high concentrations. Within the validated scope, the method demonstrates preliminary suitability for pharmaceutical quality control and therapeutic concentration monitoring; full bioanalytical validation per FDA/EMA guidance is recommended prior to pharmacokinetic applications. It is acknowledged that acetonitrile use in the plasma preparation step is included in the environmental assessments, and that long-term sustainability of the surfactant system at high concentrations warrants further evaluation.

## Introduction

Athletes are commonly susceptible to a variety of medical conditions that arise either directly from physical exertion or indirectly through compromised immunity or environmental exposure, often leading to interruptions in training^1,2^.

Amoxicillin (AMX), a β-lactam antibiotic, is widely prescribed in sports medicine due to its potent activity against both Gram-positive and Gram-negative bacteria. It acts by binding to and inhibiting penicillin-binding proteins (PBPs) —essential for cross-linking peptidoglycan chain during bacterial cell wall synthesis. AMX is frequently used to treat bacterial infections that may be exacerbated by the immunosuppressive effects of prolonged, high-intensity exercise and communal living conditions in athletic environments^3^.

In parallel, musculoskeletal injuries are prevalent in athletes, often requiring co-administration of anti-inflammatory and muscle-relaxant medications. Ibuprofen (IBU), a non-steroidal anti-inflammatory drug (NSAID), alleviates pain and inflammation by inhibiting cyclooxygenase enzymes which catalyze the conversion of arachidonic acid into pro-inflammatory prostaglandins, while chlorzoxazone (CHL) acts centrally on the spinal cord and brain stem to reduce skeletal muscle spasms. This combination therapy have complementary effects and is commonly used for managing pain, inflammation, and muscle stiffness^4–6^.

Although each of these drugs is widely prescribed individually, their concurrent use is not limited to athletes but extends to clinical scenarios involving infection-associated musculoskeletal pain, where antibiotic therapy is combined with anti-inflammatory and muscle relaxant agents. Such multidrug regimens are particularly relevant in outpatient settings and post-injury management. From, an analytical standpoint, their structural diversity — polar beta-lactam, moderately hydrophobic benzoxazolone, hydrophobic propionic acid derivative respectively— poses a genuine chromatographic challenge, making them a scientifically representative and demanding test mixture.

While modern LC methods may allow multi-analyte detection, most reported approaches are either designed for unrelated compound classes or require organic solvents and complex gradients. A critical review of the literature revealed no dedicated, validated method addressing this specific triple combination (AMX–CHL–IBU) in both pharmaceutical formulations and biological matrices under green analytical conditions. Therefore, the novelty of this work lies not only in simultaneous determination but in the development of a sustainable, micellar-based method tailored to this therapeutically relevant combination, ensuring selectivity, efficiency, and environmental compatibility.

Previous analytical efforts targeting these pharmaceuticals have primarily focused on binary combinations. Reversed-phase liquid chromatography (RP-LC)^7,8^, spectrofluorimetry^9–11^, and micellar electrokinetic chromatography (MEKC)^12^, have been developed specifically for the simultaneous analysis of CHL and IBU. Other spectrophotometric^13,14^, and chromatographic techniques^15,16^ have addressed their analysis in combination with different drugs, excluding amoxicillin (AMX). No established method exists for the simultaneous quantification of AMX, CHL, and IBU in complex biological or pharmaceutical samples.

To the best of our knowledge, the present study introduces the first validated MLC-based method for the simultaneous determination of this specific triple combination (AMX, CHL, and IBU) in both pharmaceutical dosage forms and spiked human plasma under fully organic solvent-free mobile phase conditions. While SDS/Brij-35 mixed micellar systems are established in principle, no prior validated method applies this platform to the AMX–CHL–IBU combination in these matrices simultaneously. This analytical platform not only fills a critical gap in literature but also offers a high-throughput, environmentally favorable alternative to traditional chromatographic techniques. Micellar liquid chromatography (MLC) offers distinct advantages over conventional techniques, employing low-cost, biodegradable, and environmentally safe mobile phases. The micellar system provides a dual retention mechanism, wherein hydrophilic micelle heads interact with polar compounds and hydrophobic cores retain nonpolar analytes, enabling efficient simultaneous separation of analytes with diverse polarities in a single isocratic run^17^.

Anionic sodium dodecyl sulfate (SDS) is the most widely used surfactant in MLC; however, the addition of organic solvents is typically required to improve efficiency and reduce retention times, particularly for positively charged basic compounds that tend to be strongly retained by SDS monomers adsorbed onto the stationary phase. By contrast, non-ionic surfactant Brij-35 binds to and reduces the polarity of the neutral stationary phase, significantly decreasing retention times, eliminating the need for organic solvents, this results in more sustainable, effective liquid chromatographic methods. In this study, the combination of the SDS/Brij-35 mixed micellar system was not arbitrary but based on complementary physicochemical properties. SDS, an anionic surfactant, enhances retention of neutral and hydrophobic analytes via micellar partitioning while introducing electrostatic interactions with ionizable species. Brij-35, a non-ionic surfactant, reduces excessive interaction between SDS monomers and the stationary phase, thereby improving peak symmetry and reducing retention times. The combination enables fine-tuning of selectivity through modulation of hydrophobic and electrostatic interactions, which is particularly advantageous for separating structurally diverse analytes such as AMX (polar), CHL (moderately hydrophobic), and IBU (hydrophobic acidic drug). To provide quantitative context, the CMC of SDS under the buffer conditions employed (0.01 M KH₂PO₄, pH 3.0) is approximately 1.5–3 mM^18^, well below the 150 mM concentration used, ensuring robust micelle formation throughout the analysis. Similarly, Brij-35 has a CMC of ~ 0.09 mM in aqueous media^19^, meaning the 50 mM concentration employed is approximately 500-fold above its CMC, guaranteeing effective stationary phase coating and retention time modulation. The observed elution order — AMX (tR = 1.776 min) < CHL (tR = 3.179 min) < IBU (tR = 5.627 min) — is consistent with the analytes’ log P values (0.87, 2.24, and 3.97, respectively), confirming that hydrophobic partitioning into the micellar core is the dominant retention mechanism, supplemented by electrostatic interactions between adsorbed SDS monomers and the ionizable functional groups of AMX at pH = 3. This has enabled rapid and efficient organic-solvent free liquid chromatographic separation of three structurally and chemically diverse drugs within six minutes. This streamlined, isocratic method shows potential for routine pharmaceutical quality control and, within the validated concentration range, preliminary therapeutic drug monitoring applications. Extension to full pharmacokinetic studies would require additional bioanalytical validation steps beyond the current scope.

To substantiate this potential, the method was rigorously validated according to ICH guidelines^20^, and its performance was statistically benchmarked against a previously reported methods^8,21^ using the F-ratio and Student’s t-test.

Beyond analytical performance, the method’s environmental sustainability was assessed using a comprehensive suite of green and white analytical chemistry tools, including the Analytical Eco-Scale, GAPI, AGREE, BAGI, and RGB12.

## Materials and methods

### Chemicals and reagents

Amoxicillin ((6R)-6-(α-D-(4-Hydroxyphenyl) glycylamino) penicillanic acid), Chlorzoxazone (5-Chlorobenzoxazol-2(3 H)-one) and Ibuprofen (2-(4-Isobutylphenyl) propionic acid) were generously supplied by EIPICO Pharmaceutical (Egypt).

E-MOX capsules labeled to contain 500 mg amoxicillin (EIPICO, Egypt) and Myofen capsules containing 250 mg chlorzoxazone and 200 mg ibuprofen (EVA Pharma, Egypt) were procured from a local pharmacy.

KH_2_PO_4_ and HPLC-grade methanol were procured from (Merck, Germany), Brij-35 was obtained from Alfa Aesar (USA), while sodium dodecyl sulfate (SDS) was provided by (Thermo Fischer Scientific, Germany).

Human plasma samples were obtained from the Holding Company for Biological Products and Vaccines (VACSERA), Egypt, as anonymized biological material. The study did not involve recruitment or participation of human subjects, collection of samples from donors, or access to identifiable donor information; therefore, ethical approval and informed consent were not applicable.

### Instrumentation

Chromatographic separations were performed on a Waters Alliance 2695 HPLC system equipped with an autosampler, a quaternary pump, and a photodiode array (PDA) detector (10 mm flow cell, maximum pressure of 5000 psi, the actual operating back-pressure was consistently below 1000 psi). Data were acquired and processed using Empower 3 Pro Chromatography Data Software (Version 7.41.00.00, Build 3471; Waters Corporation, Milford, MA, USA; https://www.waters.com/waters/en_US/Empower-3-Chromatography-Data-Software/nav.htm?cid=513188).

Separations were achieved on a Symmetry C18 column (75 × 4.6 mm, 3.5 μm; Waters).

The mobile phase consisted of 0.15 M sodium dodecyl sulfate (SDS), 0.05 M Brij-35, and 0.01 M potassium dihydrogen phosphate (KH₂PO₄), adjusted to pH 3.0 using a calibrated pH meter (AD1030, ADWA, Szeged, Hungary. ). It was maintained at 40 °C and delivered at a flow rate of 1.0 mL·min⁻¹. Although amoxicillin is susceptible to hydrolytic degradation under acidic conditions and elevated temperatures, the brief exposure time per run (< 6 min) and immediate analysis of freshly prepared samples minimized degradation effects; no degradation product peaks were observed in chromatograms of fresh standards. The PDA detector was set at 220 nm for analyte detection. To enhance sustainability, the mobile phase was recycled between chromatographic runs. To ensure the reliability of mobile phase recycling, system performance was monitored over consecutive injections (*n* = 10). No significant changes in retention times or peak areas were observed across all three analytes. Additionally, carryover was evaluated by injecting blank samples after high-concentration standards, confirming absence of residual peaks at analyte retention times. The system was routinely cleaned with water and subsequently purged for 15 min with a 1:1 (v/v) mixture of water and methanol to remove any adsorbed surfactants from the stationary phase.

The pH of the mobile phase was adjusted using a benchtop pH meter (AD1030, ADWA, Szeged, Hungary).

### Standard solutions

Stock solutions of AMX, CHL, and IBU (1 mg·mL⁻¹) were prepared individually by dissolving 25 mg of each drug in 25 mL of methanol. Working standard solutions (50 µg·mL⁻¹) were obtained by appropriate dilution of the stock solutions with a 50:50 (v/v) mixture of methanol and distilled water. Serial dilutions of the working solutions were then prepared to yield calibration standards at concentrations of 2.5, 5.0, 10.0, 15.0, 20.0, and 25.0 µg·mL⁻¹ for method validation.

For precision and accuracy studies, additional solutions were prepared at concentrations of 5.0, 15.0, 20.0, and 25.0 µg·mL⁻¹ for each analyte.

### Construction of calibration plots

Calibration curves for AMX, CHL, and IBU were constructed using the working solutions prepared as described in Sect. 2.3. Triplicate injections of each calibration level (2.5–25 µg·mL⁻¹) were analyzed, and the mean peak area (AUC) was plotted against concentration. Linear regression analysis was applied to obtain the calibration equations and correlation coefficients. Limits of detection (LOD) and quantification (LOQ) were calculated according to ICH guidelines.

### Analysis of pharmaceutical dosage form

The contents of five E-MOX capsules (nominal: 500 mg AMX/capsule) were pooled and weighed; a mass equivalent to one capsule was transferred into a 100 mL volumetric flask, dissolved by sonication with 80 mL methanol for 15 min, and filtered into a second 100 mL flask. The volume was completed with methanol to yield a stock solution of 5 mg/mL AMX. Independently, the contents of five Myofen capsules (nominal: 250 mg CHL + 200 mg IBU/capsule) were treated identically in a 50 mL flask to yield a stock solution of 5 mg/mL CHL and 4 mg/mL IBU. Separate stock solutions were required to maintain independent gravimetric control over each formulation’s actual content before combination. Aliquots of 5.0 mL from each stock were combined in a 250 mL volumetric flask and diluted to volume with methanol–water (50:50, v/v), yielding a combined solution of 100 µg/mL AMX, 100 µg/mL CHL, and 80 µg/mL IBU. Working solutions at 25/25/20 µg/mL and 15/15/12 µg/mL were prepared by appropriate aliquotting and dilution with mobile phase. All glassware was Class A calibrated; the three sequential dilution steps introduce a cumulative volumetric uncertainty of < 0.3%, confirmed empirically by recovery data falling within the ICH Q2(R1)-accepted accuracy range (98.0–102.0%, Table 1).

**Table 1:** Analysis results of E-MOX and Myofen capsules containing the studied medications employing the proposed HPLC method.

Pharmaceutical dosage form	Composition	Measured concentration (µg/mL)	Found % ± RSD
E-MOX	AMX	25	100.16 ± 0.167
		15	98.86 ± 0.055
Myofen	CHL	25	99.97 ± 0.254
		15	99.45 ± 0.448
	IBU	20	98.93 ± 0.149
		12	99.29 ± 0.081

### Human plasma samples

Individual 1 mL aliquots of human plasma were transferred into 15 mL centrifuge tubes and spiked with AMX, CHL, and IBU at three concentration levels (2.5, 10, and 20 µg·mL⁻¹), vortex-mixed for 1 min, and treated with 4 mL of acetonitrile to precipitate plasma proteins; the mixtures were centrifuged at 6000 rpm for 30 min, and the resulting supernatants were filtered through 0.45 μm syringe filters, after which drug concentrations were quantified using the established regression equations.

### Method validation

#### Linearity

A linear regression analysis was conducted on the calibration curves by plotting the peak area against the concentration for each tested medication. Six solutions were prepared for each drug, with concentrations ranging from 2.5 to 25 µg/mL, to achieve the required linearity.

#### LOD & LOQ

The limits of detection (LOD) and quantification (LOQ) were calculated using the slope of the calibration curve and the standard deviation (SD) of the intercept, with LOD = 3.3 SD/slope and LOQ = 10 SD/slope.

#### Accuracy

Accuracy was evaluated by analyzing nine independent samples at three concentration levels (15.0, 20.0, and 25.0 µg/mL). For each level, three replicates were prepared and analyzed, and the mean percent recovery was calculated.

#### Precision

Intraday precision (repeatability) and interday precision (reproducibility) were evaluated at three concentration levels (15.0, 20.0, and 25.0 µg/mL) for each analyte. For intraday precision, each level was analyzed in triplicate within the same day, while interday precision was assessed by repeating the analysis on three consecutive days. Precision was expressed as the relative standard deviation (%RSD) of the measured concentrations.

#### Selectivity

The selectivity of the proposed method was evaluated by analyzing two pharmaceutical dosage forms containing the three studied drugs, as well as human plasma samples spiked with the same analytes. Method performance was assessed by calculating the percent recoveries and relative standard deviation (%RSD) values, thereby confirming the ability of the method to accurately quantify the target compounds without interference from excipients or biological matrix components.

#### Robustness

The robustness of the method was examined by evaluating the consistency of peak area measurements following deliberate, minor variations in key chromatographic parameters. These included pH (3.0 ± 0.1), SDS concentration (0.15 M ± 0.01), Brij-35 concentration (0.05 M ± 0.01), detection wavelength (220 nm ± 1.0), column temperature (40 °C ± 1.0), and flow rate (1.0 mL/min ± 0.01). The method’s ability to withstand such small changes without significant impact on analytical performance was considered an indication of robustness. Acceptance criteria were defined as recovery within 98.0–102.0% and %RSD ≤ 2.0% for each parameter variation.

#### System suitability

To verify post-optimization performance and confirm the system’s reliability for the intended analysis, system suitability testing (SST) was performed as per USP standards^22^. Triplicate injections of a mixture containing 25.0 µg/mL of each drug were analyzed, and key chromatographic parameters, including resolution (Rs), selectivity factor (α), tailing factor (T), capacity factor (k´), and retention time repeatability, were calculated.

All measured parameters met the established USP acceptance limits, confirming system suitability for the intended analysis.

The current validation was conducted in accordance with ICH Q2(R1) guidelines for pharmaceutical quality control applications. Parameters including matrix effect evaluation, stability studies, and formal carryover assessment were outside the scope of this validation and are recommended for future full bioanalytical validation per FDA/EMA guidance.

### Sustainability assessment

#### Analytical eco-scale

The Analytical Eco-scale was applied to evaluate the environmental impact of the developed method. Penalty points were assigned based on four primary parameters: reagent consumption, energy requirements, waste generation, and associated hazards. The final Eco-scale score, obtained by deducting the total penalty points from an initial value of 100, provides a quantitative measure of adherence to green chemistry principles. Scores greater than 75 are considered indicative of excellent agreement with sustainable analytical practices. The detailed scoring methodology has been described previously^23^.

#### Green analytical procedure index (GAPI)

GAPI was used to evaluate the environmental impact of the analytical procedure by assessing each stage—from sample collection and preparation to final quantification. A 12-segment pictogram was generated, each segment was color-coded (green, yellow, or red) to reflect low, medium, or high environmental impact, respectively, based on predefined criteria established in the literature^24^.

#### Analytical GREEnness metric (AGREE)

The greenness of the proposed analytical method was evaluated using the Analytical GREEnness Metric (AGREE), a software-based tool developed by Pena-Pereira et al.^25^. The approach is based on the twelve principles of green analytical chemistry, which encompass operator safety, use of toxic reagents, use of renewable resources, energy consumption, analytical throughput, waste generation, use of derivatization, automation and miniaturization, number of procedural steps, proximity of the instrument to the measurement location, sample size and number of samples, and sample preparation requirements. The freely available AGREE software was employed to generate the overall greenness score and corresponding pictogram. The evaluation output is presented as a circular shaped segmented diagram, where each segment corresponds to one of the twelve principles of green analytical chemistry. Color intensity within the segments reflects the degree of compliance with each principle, while the overall greenness score is displayed at the center of the pictogram. Scores closer to 1, accompanied by a dark green coloration, indicate superior adherence to green chemistry practices.

#### Spider chart

The evaluation of the solvents employed was carried out based on five key criteria: general physicochemical properties, stability, odor, flammability, and potential health risks. Each solvent was assigned a score using standardized algorithms, with values ranging from − 5 (least favorable) to + 5 (most favorable). These scores encompassed both ecological impact and safety considerations. The results were represented as spider charts, providing a comparative graphical visualization of solvent greenness across the analytical procedures. Detailed methodology is available in a previous report^26^.

It is acknowledged that the spider chart serves as a complementary visual tool rather than a standalone quantitative metric, and its interpretation should be considered alongside the quantitative scores provided by the Eco-scale, AGREE, BAGI, and RGB12 assessments.

#### Blue applicability grade index (BAGI)

The Blue Applicability Grade Index (BAGI) was employed to evaluate the applicability and functionality of the developed analytical method. Ten parameters were considered in the assessment: sample amount, degree of automation, pre-concentration requirements, types of reagents and materials, number of samples analyzed per hour, number of samples treated simultaneously, analytical technique or instrumentation, and number of analytes determined simultaneously. Each parameter was assigned a score on a scale from 2.5 to 10, and the overall method score was calculated by summing the individual values. A BAGI pictogram was then generated, in which the cumulative score was displayed in the center and each parameter was represented by a color grade (dark blue, blue, light blue, or white) corresponding to the level of compliance^27^.

#### Whiteness assessment using RGB12 tool

The sustainability of the developed analytical procedures was evaluated using the Red-Green-Blue (RGB) model. The assessment was performed with the RGB12 algorithm, which is based on the twelve principles of White Analytical Chemistry (WAC). For this evaluation, three color-coded tables were generated: (i) a green table addressing Green Analytical Chemistry (GAC) aspects such as toxicity, waste disposal, reagent consumption, energy conservation, human health, and genetic impact; (ii) a blue table covering time and cost efficiency, as well as practical and financial feasibility; and (iii) a red table focusing on validation-related parameters, including accuracy, applicability, precision, limit of detection (LOD), and limit of quantification (LOQ). The scoring of all criteria in the three tables followed previously established methodologies^28^.

## Results and discussion

### Method optimization

**Fig. 1:**
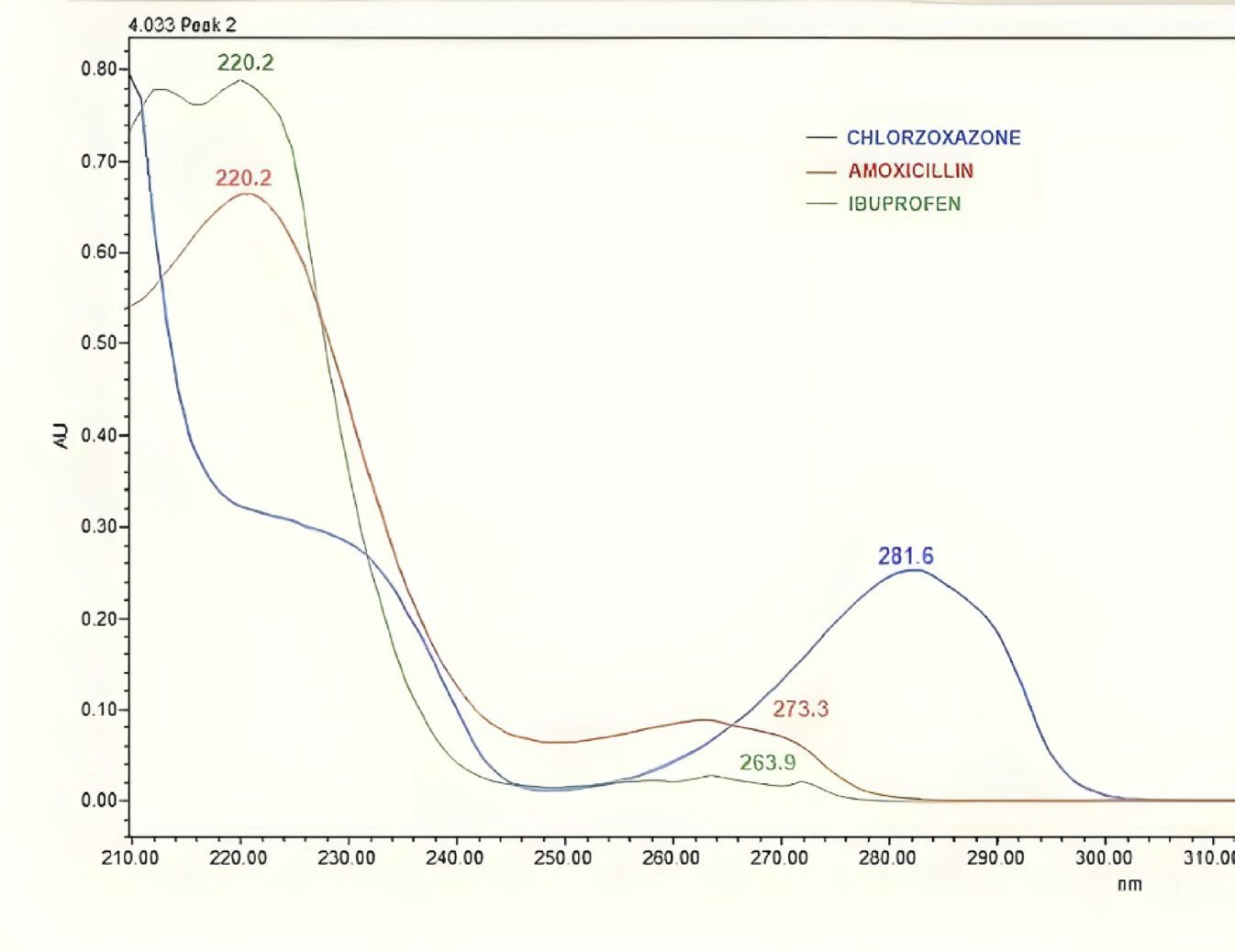
Recorded UV–Vis spectra of pure (AMX, CHL, and IBU).

**Fig. 2:**
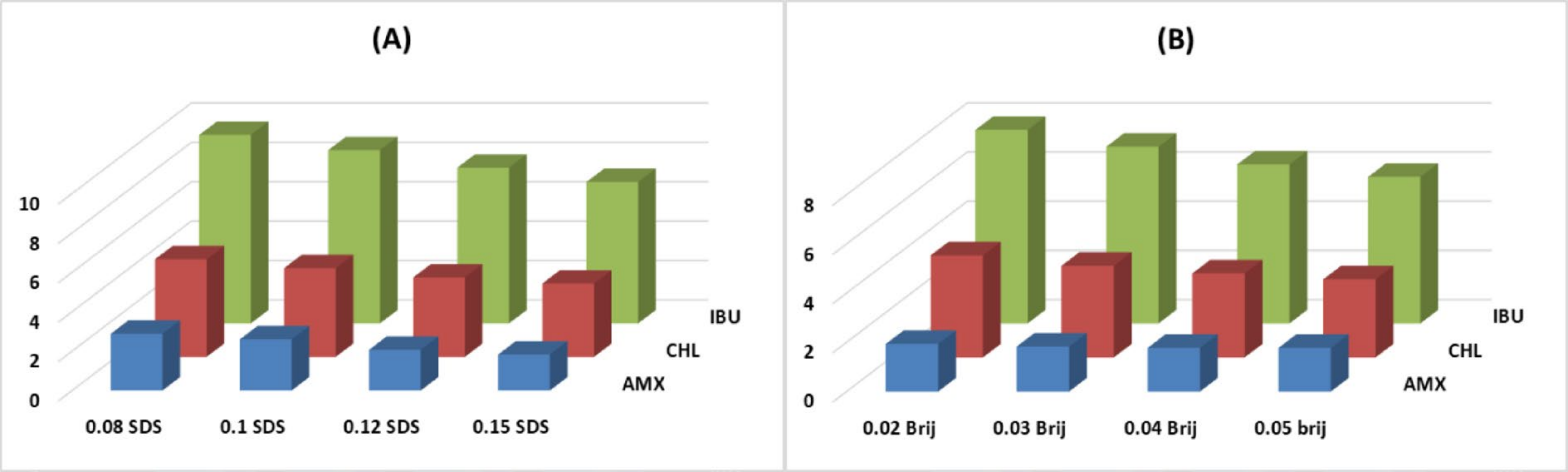
Effect of Brij-35 and SDS concentrations on the retention times of the studied drugs: (**A**) variation of SDS concentration at fixed 0.03 M Brij-35; (**B**) variation of Brij-35 concentration at fixed 0.15 M SDS.

**Fig. 3:**
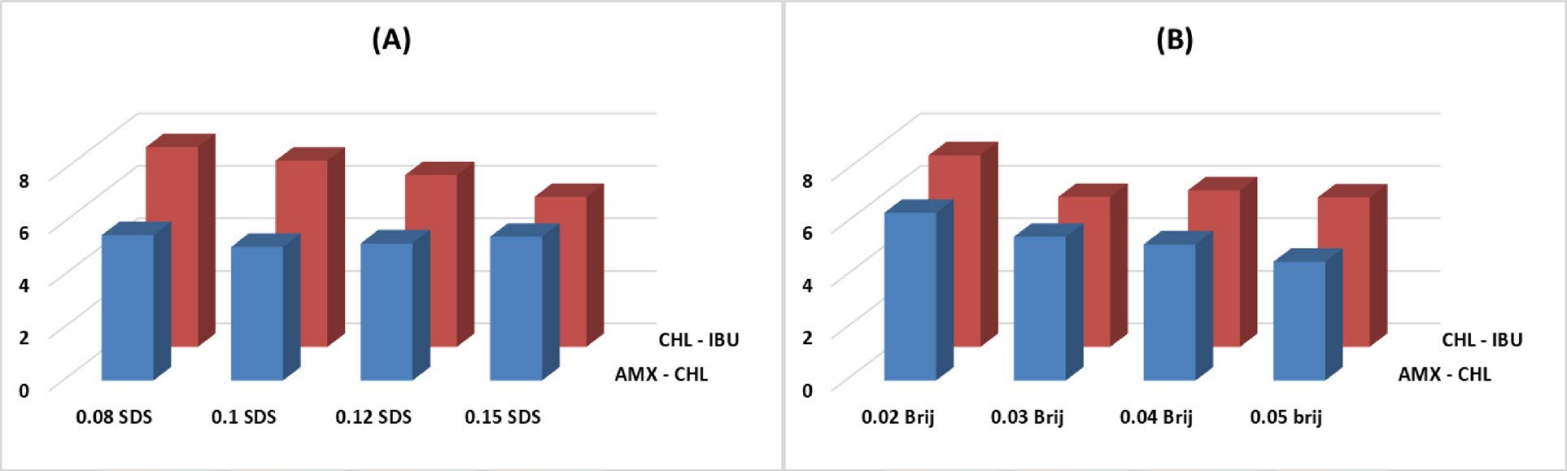
Effect of surfactant concentration on the resolution of the studied drugs: (**A**) variation of SDS at constant 0.03 M Brij-35; (**B**) variation of Brij-35 at constant 0.15 M SDS.

**Fig. 4:**
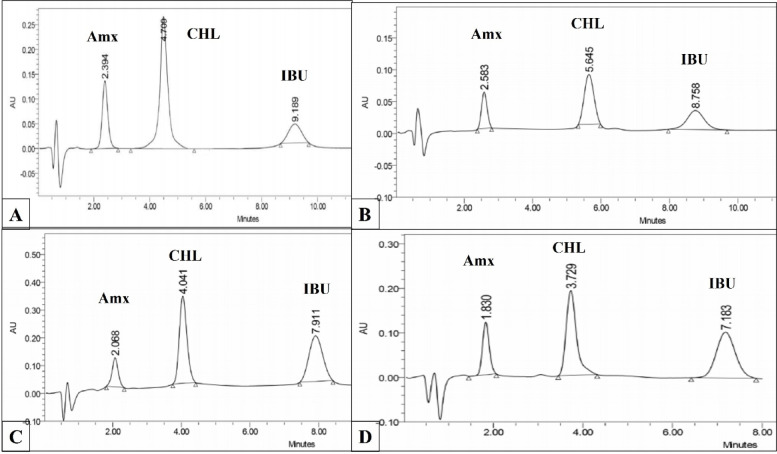
MLC chromatograms of AMX, CHL, and IBU during SDS concentration optimization at fixed 0.03 M Brij-35: (**A**) 0.08 M SDS; (**B**) 0.10 M SDS; (**C**) 0.12 M SDS; (**D**) 0.15 M SDS.

**Fig. 5:**
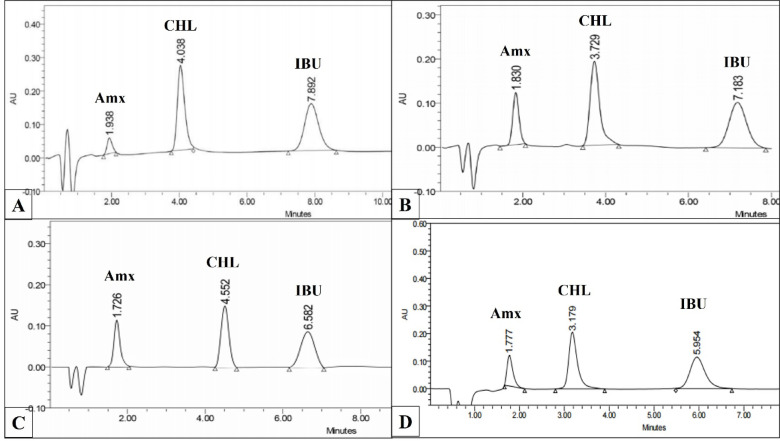
MLC chromatograms of AMX, CHL, and IBU during Brij-35 concentration optimization at fixed 0.15 M SDS: (**A**) 0.02 M Brij-35; (**B**) 0.03 M Brij-35; (**C**) 0.04 M Brij-35; (**D**) final optimized conditions confirming complete baseline separation of all three analytes within 6 min.

To determine the appropriate detection wavelength, UV–vis spectra of AMX, CHL, and IBU (25 µg·mL⁻¹ each) were recorded in the mobile phase, and strong absorbance was observed at 220 nm; this wavelength was therefore selected for subsequent analyses (Fig. 1). The selection of 220 nm as a common detection wavelength was further justified by the distinct chromophoric profiles of the three analytes: AMX absorbs at this wavelength via its β-lactam and phenolic moieties, CHL through its benzoxazolone ring system, and IBU — which lacks a strong chromophore above 220 nm — exhibits its maximum practical sensitivity at this wavelength. Thus, 220 nm represents the optimal compromise wavelength ensuring adequate and simultaneous detection sensitivity for all three structurally diverse analytes. Optimization of mobile phase pH was next investigated, taking into account the pKa values of the analytes, their ionic/neutral forms, the stability of the silica bed, and experimental trials at different pH values. A pH of 3.0 was found to provide the best resolution and peak shape. At pH 3.0, AMX (pKa 2.4, 7.4, 9.6) exists predominantly as a zwitterion with its amino group partially protonated, enabling electrostatic interactions with adsorbed SDS monomers on the stationary phase, consistent with its early elution (tR = 1.776 min) despite moderate hydrophobicity (log *P* = 0.87). CHL (pKa ~ 11.6) and IBU (pKa 4.9) are essentially unionized at this pH, making their retention governed primarily by hydrophobic partitioning into the micellar core, consistent with their log P values (2.24 and 3.97) and elution order (tR = 3.179 and 5.627 min, respectively). This pH also maintains silica bed stability, which deteriorates below pH 2.0. The effect of surfactant composition was then examined by varying the concentrations of SDS and Brij-35 and monitoring the resulting separation performance. When SDS concentration was increased from 0.08 M to 0.15 M at fixed 0.03 M Brij-35, retention times decreased progressively for all three analytes, with IBU showing the greatest sensitivity to this change (Fig. 2A). This is mechanistically consistent with IBU’s high hydrophobicity (log *P* = 3.97), which renders its retention predominantly governed by partitioning into the micellar hydrophobic core; increasing micelle concentration in the bulk mobile phase therefore shifts the partition equilibrium most strongly for IBU. By contrast, AMX (log *P* = 0.87) showed minimal retention change, as its retention at pH 3.0 is more dependent on electrostatic interactions with adsorbed SDS monomers on the stationary phase than on micellar partitioning. Increasing SDS concentration also progressively improved resolution between AMX and CHL (Fig. 3A), as the differential partitioning rates of the two analytes were amplified. When Brij-35 was varied between 0.02 and 0.05 M at fixed 0.15 M SDS, retention times decreased for all analytes due to non-ionic surfactant coating of the C18 stationary phase, which reduces its effective hydrophobicity and competitively displaces adsorbed SDS monomers (Fig. 2B). The observed plateau in AMX retention at 0.04 M Brij-35 is attributable to saturation of SDS monomer displacement at AMX-relevant interaction sites on the stationary phase, while CHL and IBU continued to elute faster as hydrophobic interactions were further attenuated (Fig. 3B). The combination of 0.15 M SDS and 0.05 M Brij-35 yielded resolution values of Rs(AMX–CHL) = 4.54 and Rs(CHL–IBU) = 5.69, both substantially exceeding the USP minimum of 2.0, confirming that this combination optimally balances selectivity and analysis time. The combination of 0.15 M SDS and 0.05 M Brij-35 at pH 3.0 provided the best balance of resolution and analysis time and was therefore selected as the optimal chromatographic condition, as evidenced by the retention time profiles (Fig. 2) and resolution values (Fig. 3) obtained across the investigated surfactant concentration ranges. Representative chromatograms obtained during the sequential optimization of SDS and Brij-35 concentrations are presented in Figs. 4 and 5, respectively. SDS concentration was optimized at a fixed Brij-35 concentration of 0.03 M across the range 0.08–0.15 M (Fig. 4, panels A–D), demonstrating a progressive reduction in retention times with increasing SDS concentration, most pronounced for IBU (tR: 9.189 → 7.183 min), consistent with its dominant hydrophobic micellar partitioning mechanism. Following selection of 0.15 M SDS as optimal, Brij-35 concentration was subsequently varied from 0.02 to 0.05 M at fixed 0.15 M SDS (Fig. 5, panels A–D), showing an overall reduction in retention times while AMX retention remained nearly unchanged — reflecting the minimal impact of Brij-35 on the electrostatically-governed retention of this analyte. Panel D of Fig. 5 (0.15 M SDS, 0.05 M Brij-35) represents the final optimized mobile phase composition, confirming complete baseline separation of all three analytes within 6 min with Rs(AMX–CHL) = 4.54 and Rs (CHL–IBU) = 5.69. It is acknowledged that the univariate one-factor-at-a-time optimization strategy employed, while theory-guided and systematic, does not capture potential parameter interactions that a multivariate Design of Experiments approach — such as central composite or Box-Behnken design — would provide. This represents a methodological limitation, and DoE-based optimization is recommended for future refinement of the method.

**Fig. 6:**
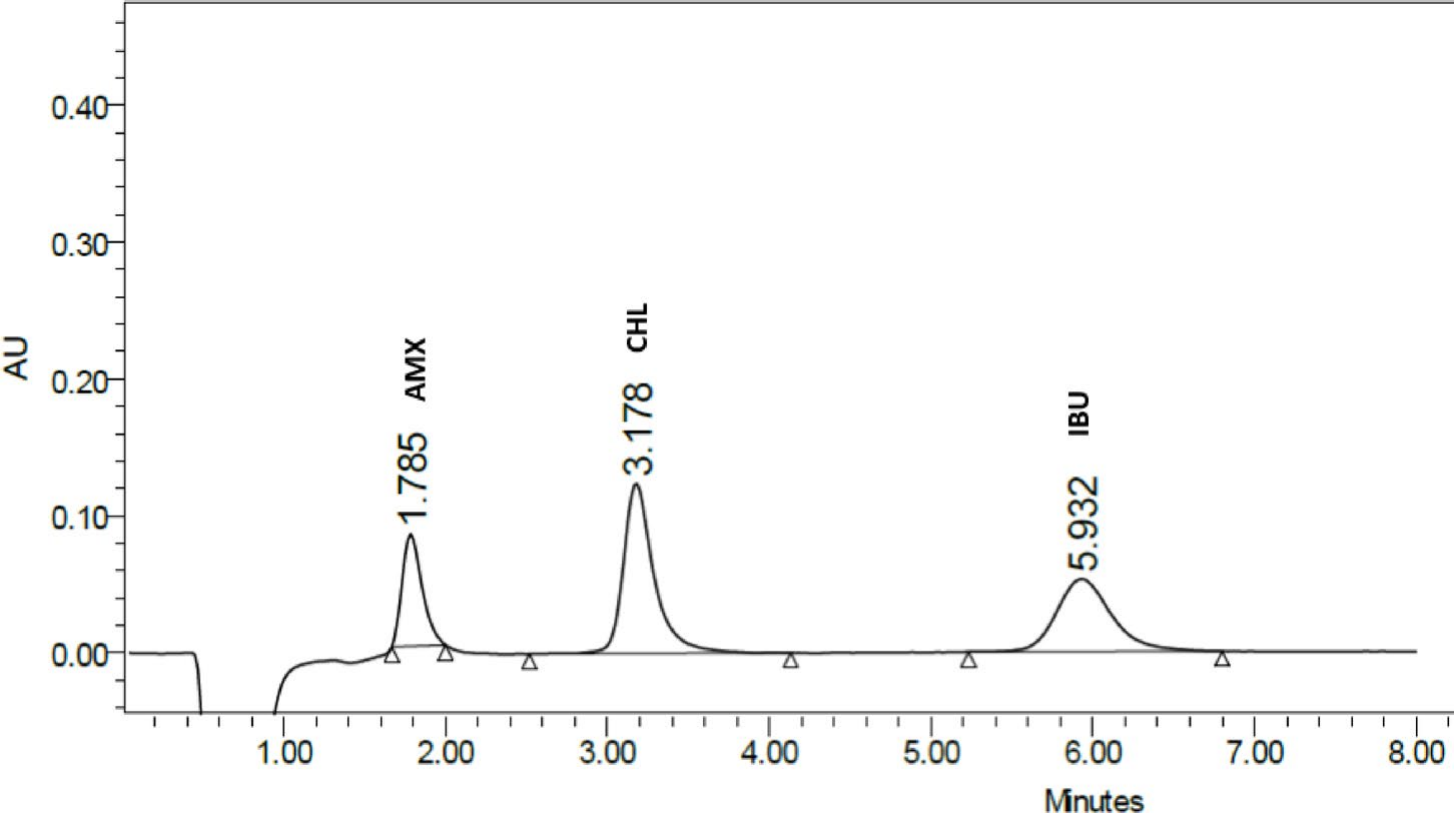
MLC chromatogram of a resolved mixture containing 15 µg/mL AMX, 15 µg/mL CHL, and 12 µg/mL IBU from E-MOX and Myofen capsules.

Calibration curves were prepared as described in the experimental section and analyzed using a linear regression model (Table 2). The developed method was successfully applied for the simultaneous determination of AMX, CHL, and IBU in their freely combined pharmaceutical formulations, with results presented in Table 1. The method demonstrated excellent performance, accurately quantifying both drugs without any noticeable interference from additives or excipients, as confirmed by the absence of co-eluting peaks (Fig. 6).

**Table 2:** Validation parameters and system suitability results for the proposed HPLC method.

Parameters	Drug name		
	AMX	CHL	IBU
Linearity (µg/mL)	2.5–25	2.5–25	2.5–25
Linearity equation	y = 42015x − 8859.9	y = 114392x − 56239	y = 109750x − 53695
R2	0.9993	0.9991	0.9997
LOD (µg/mL)	0.664	0.744	0.435
LOQ (µg/mL)	2.011	2.253	1.317
Flow rate (mL/min)	1	1	1
Retention time (min)	1.776	3.179	5.954
Resolution (Rs) (> 2)	–	4.54	5.69
Selectivity factor (α) (≥ 1)	–	2.795	2.271
Tailing factor (T) (≤ 2)	1.38	1.45	1.22
Capacity factor (k′) (> 1)	0.78	2.18	4.95

### Method validation

The suggested approach was validated using the ICH guidelines^9^. The approach was validated by taking into account the following criteria:

#### Linearity

**Fig. 7:**
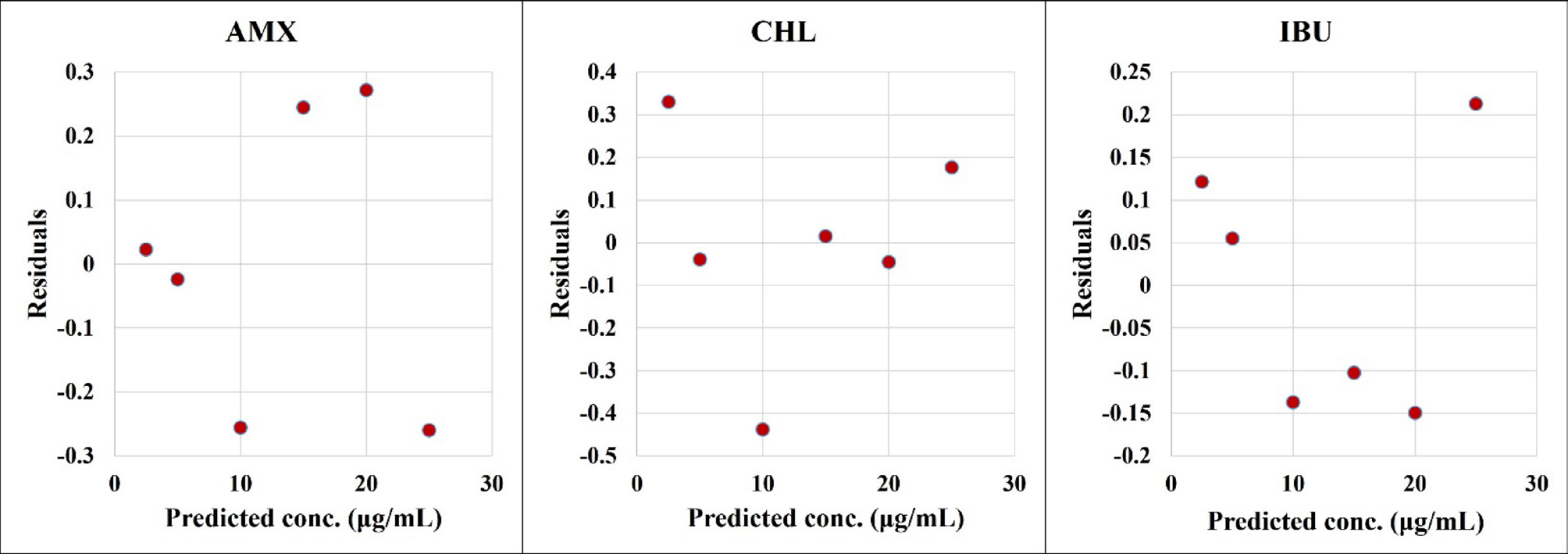
Residual plots for the predicted concentrations of AMX, CHL, IBU using the proposed method.

A clear linear correlation was observed between peak area and analyte concentration, as evidenced by linear least squares regression analysis. The regression equations and statistical parameters for AMX, CHL, and IBU are presented in Table 2. All analytes demonstrated linearity within the studied concentration ranges, with R² values spanning 0.9991–0.9997. Residual analysis confirmed random distribution of residuals without systematic bias across all concentration levels (Fig. 7), supporting the appropriateness of the linear model.

#### Limits of detection (LOD) and limits of quantitation (LOQ)

The developed method demonstrated adequate sensitivity for the intended pharmaceutical and plasma monitoring application, with LOD values of 0.44–0.74 µg/mL and LOQ values of 1.32–2.25 µg/mL, (Table 2). These values are below the reported therapeutic Cmax values for all three analytes (AMX: 5.9 µg/mL; CHL: 3.45 µg/mL; IBU: 18 µg/mL), confirming preliminary analytical suitability for therapeutic concentration monitoring within the validated range; extension to full pharmacokinetic profiling would require further sensitivity optimization and bioanalytical validation.

It is acknowledged that the LOQ values reported are suitable for quantification within the therapeutic Cmax range and are therefore appropriate for therapeutic drug monitoring and pharmaceutical quality control purposes. Extension to lower concentration ranges, as required for full pharmacokinetic profiling during elimination phases, would necessitate further sensitivity optimization.

#### Accuracy

The proposed method demonstrated excellent accuracy, as reflected by the mean recovery percentages and associated standard deviations for the three analytes. Recovery values were consistently close to 100%, with AMX ranging from 98.59% to 99.22%, CHL from 100.37% to 100.51%, and IBU from 98.82% to 101.10%, confirming the trueness of the method across the tested concentration levels, (Table 3).

**Table 3:** Accuracy and precision of AMX, CHL, and IBU determination using the proposed HPLC method.

Drug	Conc (µg/mL)	Accuracy (%R ± SD)	Intraday precision (RSD)	Inter-day precision (RSD)
AMX	25	99.218 ± 0.658	1.599	0.879
	20	98.585 ± 0.386	1.546	0.572
	15	99.189 ± 0.461	0.788	0.759
CHL	25	100.371 ± 0.969	0.517	1.424
	20	100.373 ± 0.851	0.406	0.187
	15	100.509 ± 0.547	0.398	0.344
IBU	25	98.815 ± 0.422	0.357	0.745
	20	99.264 ± 0.768	0.754	0.625
	15	101.098 ± 0.238	0.398	0.799

#### Precision

The precision of the proposed method was confirmed by the low relative standard deviation (RSD%) values obtained for AMX, CHL, and IBU at different concentration levels (Table 3). Intra-day RSD values were consistently below 1.60%, while inter-day RSD values did not exceed 1.42%, demonstrating excellent repeatability and reproducibility of the method.

#### Selectivity

**Fig. 8:**
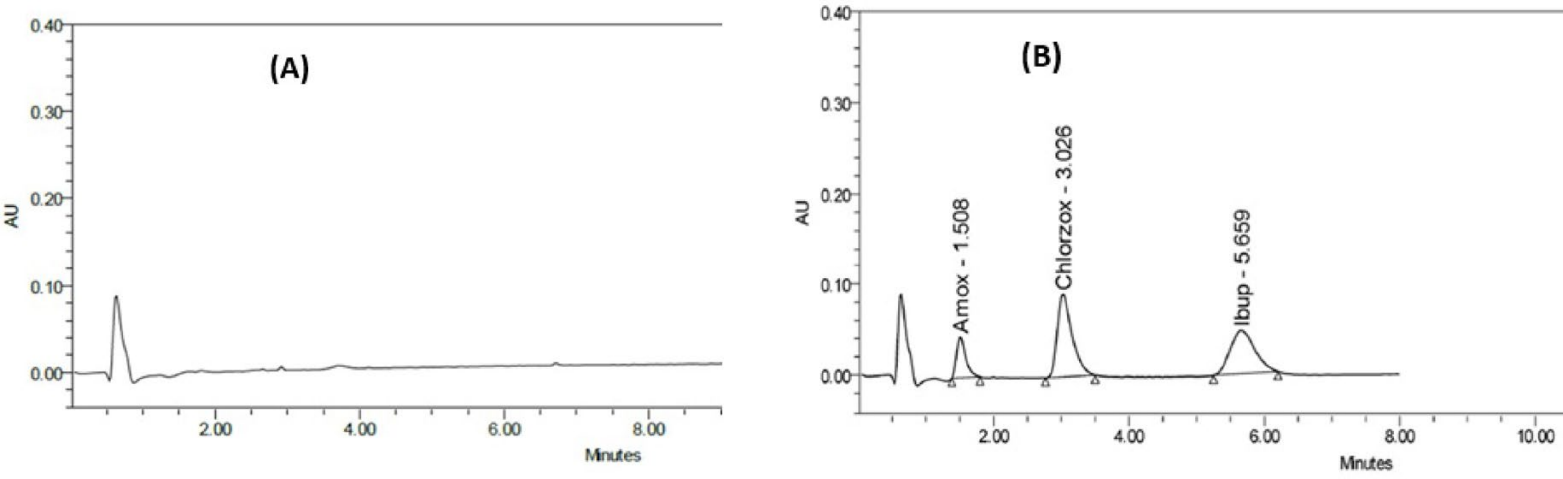
MLC chromatograms of (**A**) blank human plasma and (**B**) plasma spiked with 10 µg/mL each of AMX, CHL, and IBU.

Selectivity was evaluated by comparing chromatograms of blank matrices (excipient solution and drug-free human plasma) with those of the corresponding spiked samples. No interfering peaks were detected at the retention times of AMX (tR = 1.776 min), CHL (tR = 3.179 min), or IBU (tR = 5.954 min) in either the pharmaceutical excipient matrix or the plasma matrix, confirming that co-eluting matrix components do not contribute to the measured analyte signals (Figs. 6 and 8). Chromatographic resolution between adjacent analyte pairs was Rs(AMX–CHL) = 4.54 and Rs(CHL–IBU) = 5.69, both substantially exceeding the USP acceptance criterion of Rs ≥ 2.0, confirming complete baseline separation. The method demonstrated excellent accuracy in pharmaceutical matrices, with recoveries of 98.86–100.16% and %RSD of 0.06 − 0.45% (Table 1). In spiked human plasma, mean recoveries of 98.43–101.59% with standard deviations consistently below 0.58 were obtained (Table 4), confirming selectivity and quantitative reliability in the biological matrix.

**Table 4:** Determination of the studied medications in spiked human plasma samples employing the proposed HPLC method.

Parameter	AMX		CHL		IBU	
	Taken (µg/mL)	Found (%)	Taken (µg/mL)	Found (%)	Taken (µg/mL)	Found (%)
	2.5	102.26	2.5	98.25	2.5	97.9
	10	101.29	10	99.22	10	98.58
	20	101.22	20	98.59	20	98.8
Mean ± SD		101.59 ± 0.581		98.69 ± 0.492		98.43 ± 0.469

#### Robustness

The robustness of the proposed HPLC method was confirmed by evaluating its performance under deliberate, minor variations in chromatographic parameters (Table 5). Adjustments in column temperature (39–41 °C), flow rate (0.99–1.01 mL/min), mobile phase pH (2.9–3.1), surfactant concentrations (0.14–0.16 M SDS with 0.04–0.06 M Brij), and detection wavelength (219–221 nm) produced no significant effect on the assay of the three analytes. Across all tested conditions, percentage recoveries remained within 98.98–101.22%, while %RSD values did not exceed 1.82%. These results demonstrate the method’s ability to tolerate small but deliberate variations in operating conditions without compromising accuracy or precision, thereby attesting to its robustness. The chromatographic parameters reported under system suitability (Table 2) — including tR, Rs, and k′ at the nominal optimal conditions — serve as the reference baseline for robustness evaluation. The stability of %recovery and %RSD across all tested perturbations (Table 5) is consistent with negligible changes in these chromatographic parameters, as the tested variation ranges (± 0.1 pH unit, ± 0.01 M surfactant, ± 1 °C, ± 0.01 mL/min, ± 1 nm) are well within the method’s demonstrated tolerance.

**Table 5:** Evaluation of robustness parameters for the proposed HPLC method.

Parameter variation		$\:\%\:{recovery}^{a}\:\:\pm\:\:RSD$		
		AMX	CHL	IBU
Temperature (C°)	39	99.33 ± 0.561	98.98 ± 0.169	99.08 ± 0.463
	40	99.01 ± 0.795	100.44 ± 0.749	100.92 ± 0.23
	41	99.63 ± 0.403	99.75 ± 0.256	100.92 ± 0.388
Flow rate (mL/min)	0.99	99.11 ± 0.447	99.03 ± 0.354	98.77 ± 0.942
	1	99.01 ± 0.795	100.44 ± 0.749	100.92 ± 0.23
	1.01	100.02 ± 0.187	99.69 ± 1.158	101.22 ± 1.051
Ph	2.9	99.68 ± 0.51	100.12 ± 0.585	100.75 ± 0.873
	3	99.01 ± 0.795	100.44 ± 0.749	100.92 ± 0.23
	3.1	99.77 ± 0.436	99.34 ± 1.188	99.96 ± 1.823
Concentration of SDS and Brij (M)	0.14 M SDS, 0.04 M Brij	99.83 ± 0.58	99.56 ± 1.204	99.32 ± 0.93
	0.15 M SDS, 0.05 M Brij	99.01 ± 0.795	100.44 ± 0.749	100.92 ± 0.23
	0.16 M SDS, 0.06 M Brij	99.68 ± 0.446	100.69 ± 0.037	100.75 ± 0.873
Wavelength (nm)	219	100.23 ± 0.174	99.59 ± 1.164	101.1 ± 1.032
	220	99.01 ± 0.795	100.44 ± 0.749	100.92 ± 0.23
	221	99.84 ± 0.544	100.37 ± 1.09	99.16 ± 1.544

^a^ Mean of three determinations.

#### System suitability testing

System suitability parameters were evaluated according to USP guidelines based on triplicate injections of a mixture containing 25.0 µg/mL of each analyte. Resolution values (Rs) exceeded the acceptance criterion of 2.0, confirming adequate separation of all adjacent analyte pairs: Rs(AMX–CHL) = 4.54 and Rs(CHL–IBU) = 5.69 (Table 2), both indicating excellent baseline resolution. The selectivity factors (α) were well above 1.0, reflecting strong discriminatory power between analytes. Peak symmetry was within acceptable limits, with tailing factors (T) of 1.38, 1.45, and 1.22 for AMX, CHL, and IBU respectively, all below the USP limit of 2. Capacity factors (k′) were 0.78, 2.18, and 4.95 for AMX, CHL, and IBU respectively. The k′ for AMX (0.78) is noted to be slightly below the generally recommended minimum of k′ > 1.0; however, as demonstrated by Rs(AMX–CHL) = 4.54 and validated recoveries of 98.59–99.22% (Table 3), this does not compromise analytical performance.

Collectively, these parameters confirm that the optimized method fulfills system suitability requirements and provides reliable chromatographic performance for simultaneous analysis of the three drugs.

Representative chromatograms demonstrating system suitability performance are provided in Fig. 6.

### Applications

#### Application to pharmaceutical dosage forms

The applicability of the proposed method was evaluated by analyzing commercial capsule formulations of E-MOX (AMX) and Myofen (CHL and IBU) (Table 1). The method achieved accurate quantification of all analytes without interference from excipients, as indicated by recoveries of 98.86–100.16% for AMX, 99.45–99.97% for CHL, and 98.93–99.29% for IBU. Precision was also confirmed, with %RSD values not exceeding 0.45%. These results demonstrate that the developed procedure is both accurate and precise, and therefore suitable for routine analysis of the studied drugs in their pharmaceutical dosage forms. All recovery values obtained from pharmaceutical dosage form analysis fell within the ICH Q2(R1)-accepted accuracy range of 98–102%, with %RSD ≤ 0.45%, confirming compliance with labeled claims for all three analytes.

#### Application to spiked human plasma

Following a simple protein precipitation protocol, the developed approach was effectively applied for the determination of AMX, CHL, and IBU in spiked human plasma samples. The method demonstrated reliable performance at concentration levels as low as 2.5 µg/mL, which is well below the reported therapeutic Cmax values for the three analytes. Specifically, the maximum plasma concentrations following oral administration were previously reported as 5.9 µg/mL for AMX (500 mg dose)^29^, 3.45 ± 0.84 µg/mL for CHL (250 mg dose)^30^, and 18 µg/mL for IBU (200 mg dose)^31^. The established sensitivity thus enables accurate quantification of these drugs within clinically relevant ranges.

As shown in Table 4, the proposed method achieved mean recovery values of 101.59 ± 0.581% for AMX, 98.69 ± 0.492% for CHL, and 98.43 ± 0.469% for IBU across the tested concentration levels (2.5, 10, and 20 µg/mL). These results confirm the high accuracy and trueness of the method. The recovery mean percentages consistently ranged between 97.9 and 102.26%, while the low standard deviations reflect the excellent precision of the assay.

Importantly, the absence of significant matrix interference was evident, as the recoveries remained consistent across all studied concentrations with low variability. Blank injections performed between samples confirmed the absence of carryover. Formal matrix effect and stability assessments were not conducted in the current study and are recommended for future full bioanalytical validation per FDA/EMA guidance; however, the consistency of recovery values across concentration levels (97.90–102.26%) provides indirect evidence of negligible matrix interference under the MLC conditions employed.

It is acknowledged that the use of Cmax reference values serves solely to confirm analytical suitability within the therapeutic monitoring range and does not imply full pharmacokinetic validation. Additionally, the plasma selectivity assessment was conducted using spiked pooled human plasma from healthy volunteers; evaluation in hemolyzed or lipemic plasma samples was beyond the scope of the current work. Real sample analysis, extended pharmacokinetic range validation, and assessment across different plasma conditions including hemolyzed and lipemic matrices are recommended for future work.

### Statistical comparison

The performance of the proposed method was statistically evaluated against previously reported reference procedures^8,21^ for the determination of AMX, CHL, and IBU in their pure forms. The results, summarized in Table 6, demonstrate excellent agreement between the proposed and reference methods.

**Table 6:** Statistical comparison of the proposed and reported methods for the analysis of AMX, CHL, and IBU in pure form.

Parameters	Proposed method			Reported method^21^	Reported method^8^	
	AMX	CHL	IBU	AMX	CHL	IBU
Mean	99.204	100.441	100.181	99.91	99.89	100.12
± SD	0.56	0.70	0.50	0.32	0.65	0.71
Variance	0.31	0.49	0.25	0.10	0.42	0.5
n	6	6	6	3	6	6
t-test	1.78	1.26	1.01	–	–	–
	2.365^*^	2.228^*^	2.228^*^			
F-ratio	3.06	1.16	0.17	–	–	–
	5.79^*^	5.05^*^	5.05^*^			

(*) Shows the t-test and F-ratio tabulated results at *P* = 0.05.

The mean recovery values obtained by the proposed method (99.20% for AMX, 100.44% for CHL, and 100.18% for IBU) were in close concordance with those of the reported methods (99.91%, 99.89%, and 100.12%, respectively). Statistical analysis confirmed that the calculated t-values (1.78 for AMX, 1.26 for CHL, and 1.01 for IBU) were all lower than the corresponding tabulated values at *P* = 0.05 (2.365, 2.228, and 2.228, respectively), indicating no significant differences in accuracy.

Similarly, the calculated F-ratios (3.06, 1.16, and 0.17 for AMX, CHL, and IBU, respectively) did not exceed the critical values (5.79, 5.05, and 5.05 at *P* = 0.05), confirming that the precision of the proposed method was statistically comparable to that of the reported ones.

A summary comparison of the proposed method with previously reported procedures in terms of run time, linearity range, LOD values, mobile phase composition, and detection wavelength is presented in Table 7. The proposed method demonstrates superior analysis speed (6 min vs. 8–15 min), comparable or improved sensitivity for AMX and IBU, and a fully organic solvent-free mobile phase — the only such procedure among those compared. While one reported method achieved lower LOD values for CHL and IBU, it employed toxic organic solvents, covered a narrower linearity range, and did not address the AMX–CHL–IBU combination. The proposed method is the only validated procedure simultaneously quantifying all three analytes in both pharmaceutical and biological matrices under green analytical conditions. It is acknowledged that hyphenated techniques such as LC-MS/MS offer superior sensitivity for individual analyte determination; however, the proposed method was deliberately developed as a green, cost-effective, and instrumentally accessible alternative for routine pharmaceutical quality control and therapeutic drug monitoring. This is particularly relevant in resource-limited laboratory settings where advanced hyphenated instrumentation is not routinely available, including many analytical laboratories in developing countries. The selected reference methods therefore represent the most appropriate published comparators for the intended application scope and instrumentation context.

**Table 7:** Comparison of analytical performance parameters of the proposed method with reported methods.

Parameter	Run time (min)	Linearity range (µg/mL)	LOD (µg/mL)			Mobile phase	Detection wavelength (nm)
			AMX	CHL	IBU		
Proposed method	6	2.5–25	0.663	0.744	0.435	SDS, Brij-35	220
Reported method^8^	12	2.5–250	–	0.47	0.71	Acetonitrile, triethylamine	226
Reported method^16^	15	2–10	–	0.02	0.04	Acetonitrile, triethylamine	215
Reported method^21^	8	10–30	1.545	–	–	Acetonitrile, methanol, phosphoric acid	210

### Sustainability assessment

**Fig. 9:**
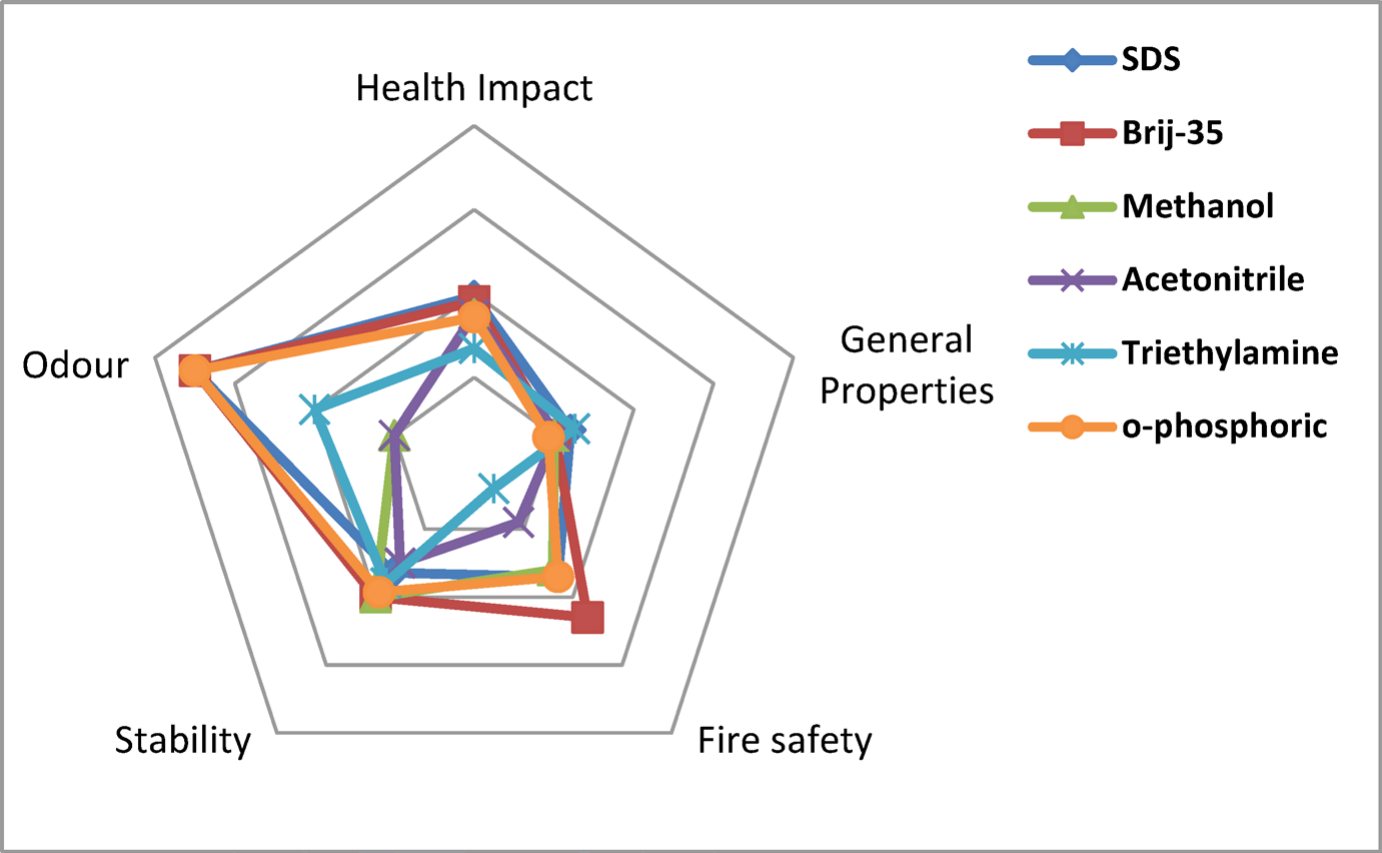
Primary spider chart for SDS, Brij-35, Methanol, Acetonitrile, Triethylamine, and o-phosphoric acid.

**Fig. 10:**
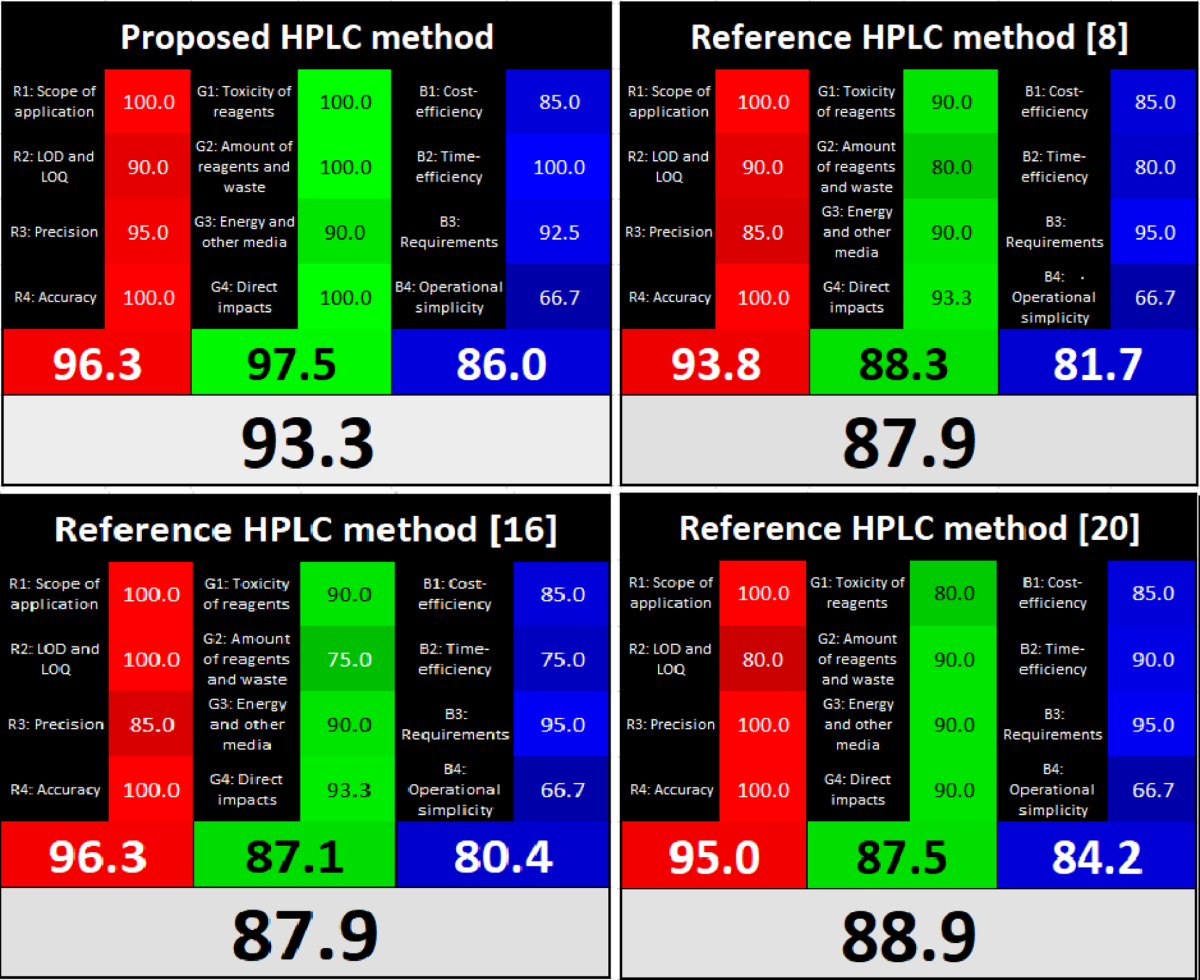
Whiteness assessment (RGB12 tool) of the proposed HPLC method compared with the reported methods^8,16,21^.

Five complementary sustainability tools were applied^8,16,21^, each addressing a distinct dimension: Eco-scale (reagent/waste penalty scoring), GAPI (stage-by-stage procedural impact), AGREE (12 GAC principles), BAGI (applicability and throughput), and RGB12 (integrated accuracy, efficiency, and greenness). Comparative results are summarized in Tables 7 and 8; Figs. 9 and 10.

**Table 8:** Eco-scale assessment of the proposed HPLC method compared with reported methods.

Category	Method				
	Proposed method	Reported method^8^	Reported method^16^		Reported method^21^
1. Chemicals	Amount x hazard type x hazard amount				
SDS	1 × 2 × 2				
Brij-35	1 × 1 × 1				
Methanol					1 × 2 × 3
Acetonitrile		1 × 2 × 2	2 × 2 × 2		1 × 2 × 2
Triethylamine		1 × 2 × 3	2 × 3 × 2		
O-Phosphoric acid					1 × 2 × 2
2. Occupational hazard	Emission of vapors and gases to the air = 3				
3. Energy (kWh/sample)	≤ 1.5 = 1				
4. Waste					
a. Waste amount (mL)	1–10 = 3	> 10 = 5	> 10 = 5		1–10 = 3
b. Waste treatment	Biodegradation = 1	No treatment = 3	No treatment = 3		No treatment = 3
Total penalty points	13	22	32		24
ECO scale score	87	78	68		76

#### Eco-scale assessment

It should be noted that while SDS is readily biodegradable under aerobic conditions, Brij-35, a non-ionic polyethoxylate surfactant, exhibits greater resistance to biodegradation and may persist in aquatic environments under anaerobic conditions; this limitation is not fully captured by standard greenness scoring tools. Additionally, acetonitrile (4 mL per plasma sample) is employed exclusively for protein precipitation and is explicitly included in the Eco-scale penalty scoring. Waste treatment by controlled biodegradation or adsorption-based removal is therefore recommended prior to disposal, and long-term sustainability of the surfactant system at high concentrations warrants further evaluation.

Within these acknowledged boundaries, the proposed method achieved an Eco-scale score of 87 (penalty score: 13), reflecting a comparatively favorable environmental profile relative to the reference methods (scores: 78, 68, and 76; Table 8). The mobile phase itself is entirely organic solvent-free, composed solely of aqueous SDS, Brij-35, and phosphate buffer, which distinguishes the chromatographic system favorably from conventional acetonitrile- or methanol-based RP-HPLC procedures.

#### GAPI assessment

The GAPI pictogram (Table 9, fifth row) shows predominant green and yellow zones with minimal red areas, indicating low environmental impact across all procedural stages, in contrast to the higher red proportions observed for the reference methods.

**Table 9:** Sustainability assessment of the proposed method versus reported methods using AGREE, GAPI, and BAGI tools.

Technique	Proposed method	Reported method^8^	Reported method^16^	Reported method^21^
	MLC (RP-HPLC–UV)	RP-HPLC–UV	RP-HPLC–UV	RP-HPLC–UV
Mobile phase composition	Isocratic elution using a mixture of 0.15 M SDS, 0.05 M Brij-35, and 0.01 M KH2PO4 at PH 3	Gradient elution using 0.2% aqueous triethylamine (adjusted to pH 5.1) and acetonitrile.	A mixture of acetonitrile: 0.2% triethylamine (adjusted to pH 3.2).	an aqueous solution of Acetonitrile, Methanol, and 0.2 M phosphoric acid within ratio of (30: 30: 40) adjusted to pH 3
Analyte similarity	AMX, CHL, and IBU	CHL and IBU	CHL and IBU	AMX
Run time	6 min	12 min	15 min	8 min
GAPI assessment				
AGREE assessment				
BAGI assessment				

#### AGREE assessment

The proposed method scored 0.77 on the AGREE radial diagram (Table 9, sixth row), outperforming the reference methods (0.55, 0.54, and 0.64), reflecting minimal solvent consumption, safer reagents, short run time, and low energy demand.

#### Spider chart

The spider chart (Fig. 9) confirms the favorable profile of the solvent system. SDS, Brij-35, and methanol scored highly across health, stability, and fire safety categories, while acetonitrile and triethylamine used in reference methods clustered toward lower values, reflecting higher toxicity and environmental risk.

#### Blueness assessment

The proposed method achieved a BAGI score of 80 (Table 9, seventh row), exceeding the reference methods (77.5), reflecting a marginally higher applicability score attributed to isocratic elution, simultaneous three-analyte determination, and absence of organic solvent in the mobile phase, as scored by the BAGI framework.

#### Whiteness assessment

The RGB12 evaluation (Fig. 10) yielded an overall score of 93.3 for the proposed method, with scores of 97.5 (greenness), 96.3 (accuracy), and 86.0 (efficiency), compared to overall scores of 87.9, 87.9, and 88.9 for the reference methods.

## Conclusion

In this work, a validated micellar HPLC method was developed for the simultaneous determination of amoxicillin, chlorzoxazone, and ibuprofen in bulk materials, pharmaceutical dosage forms, and spiked human plasma, filling an identified gap in the analytical literature for this specific therapeutic combination. The method provides rapid separation within 6 min using an organic solvent-free mobile phase, with confirmed linearity, accuracy, precision, and selectivity in both pharmaceutical and plasma matrices. Comprehensive greenness assessments (Eco-scale, GAPI, AGREE, BAGI, and RGB12) demonstrated a comparatively favorable environmental profile relative to conventional RP-HPLC procedures using acetonitrile/triethanolamine mobile phases. It is acknowledged that acetonitrile is employed in the plasma preparation step and that standard greenness metrics do not fully account for the environmental persistence of non-ionic surfactants such as Brij-35 at high concentrations; these limitations have been transparently incorporated into the assessment. Furthermore, limitations include a relatively narrow calibration range and simplified protein precipitation that may require augmentation with additional sample pre-treatment steps, such as liquid-liquid extraction or solid-phase extraction, for more complex biological matrices and future pharmacokinetic applications per FDA/EMA bioanalytical guidance. Notably, the absence of an internal standard represents an acknowledged limitation for plasma analysis; while the consistent extraction recoveries obtained (98.43–101.59%) demonstrate minimal extraction variability within the current scope, incorporation of a structurally analogous internal standard — such as a deuterated analog or structurally related compound — is explicitly recommended for future pharmacokinetic applications to further compensate for extraction variability and enhance quantitative reliability. Additionally, the plasma selectivity assessment was conducted using spiked pooled human plasma from healthy volunteers; evaluation in hemolyzed or lipemic plasma samples was beyond the scope of the current work. Future work should incorporate a structured internal standard, more comprehensive clean-up procedures, extended stability studies, expanded pharmacokinetic range validation, and assessment of method performance across different plasma conditions including hemolyzed and lipemic matrices.

## Data Availability

All data generated or analyzed during this study are available on request.
